# Exposure to Fine Particulate Matter (PM2.5) and Heavy Metals During the Second Trimester of Pregnancy Increases the Risk of Preeclampsia and Eclampsia: An Analysis of National Health Insurance Claims Data from South Korea

**DOI:** 10.3390/medicina61071146

**Published:** 2025-06-25

**Authors:** Kuen Su Lee, Won Kee Min, Yoon Ji Choi, Jeongun Cho, Sang Hun Kim, Hye Won Shin

**Affiliations:** 1 Department of Anesthesiology and Pain Medicine, Eulji University Uijeongbu Eulji Medical Center, Eulji University School of Medicine, Uijeongbu 11759, Republic of Korea; dlrmstnx@eulji.ac.kr; 2 Department of Anesthesiology and Pain Medicine, Korea University Ansan Hospital, Korea University College of Medicine, Ansan 15355, Republic of Korea; wonkeemin@gmail.com (W.K.M.); migag4@naver.com (J.C.); 3 Department of Anesthesiology and Pain Medicine, Chosun University, Chosun University Hospital, Gwangju 61453, Republic of Korea; ksh3223@chosun.ac.kr; 4 Department of Anesthesiology and Pain Medicine, College of Medicine, Korea University Anam Hospital, Seoul 02841, Republic of Korea; hwshin@korea.ac.kr

**Keywords:** air pollutants, heavy metals, preeclampsia

## Abstract

*Background and Objectives*: Air pollutants have been shown to affect hypertensive disorders and placental hypoxia due to vasoconstriction, inflammation, and oxidative stress. The objective of this study was to evaluate whether high levels of maternal exposure to heavy metals during the second trimester of pregnancy are associated with an increased risk of preeclampsia and eclampsia, using national health insurance claim data from South Korea. *Methods:* Data on mothers and their newborns from 2016 to 2020, provided by the National Health Insurance Service, were used (*n* = 1,274,671). Exposure data for ambient air pollutants (PM2.5, CO, SO_2_, NO_2_, and O_3_) and heavy metals (Pb, Cd, Cr, Cu, Mn, Fe, Ni, and As) during the second trimester of pregnancy were retrieved from the Korea Environment Corporation. Atmospheric condition data based on the mother’s registration area were matched. A logistic regression model was adjusted for maternal age, infant sex, season of conception, and household income. *Results:* In total, 16,920 cases of preeclampsia and 592 cases of eclampsia were identified. In the multivariate model, copper exposure remained significantly associated with an increased risk of preeclampsia (odds ratio: 1.011; 95% confidence interval: 1.001–1.023), and higher ozone exposure during pregnancy was associated with an elevated risk of eclampsia. *Conclusions:* Increased copper exposure during the second trimester of pregnancy was associated with a high incidence of preeclampsia.

## 1. Introduction

Preeclampsia is a hypertensive disorder of pregnancy, accounting for approximately 45% of diagnoses of hypertensive disorders during pregnancy [1]. Preeclampsia is a complex syndrome affecting approximately 3–5% of all pregnant women [2,3]. It typically presents in pregnant women after 20 weeks of gestation with elevated maternal blood pressure or proteinuria [4]. Preeclampsia is often accompanied by dysfunction in multiple organs, including the liver, kidneys, circulatory system, and placenta [5,6]. It may lead to thrombocytopenia and hemolysis, potentially progressing to HELLP syndrome. Nervous system manifestations encountered are headache, blurred vision, scotomata, and hyperreflexia. Although uncommon, posterior reversible encephalopathy syndrome may accompany severe preeclampsia and is associated with a poor prognosis if diagnosis and treatment are delayed [7]. It is a pregnancy-associated complication for which there is no effective treatment other than delivery, and it can potentially lead to life-threatening complications for both the mother and fetus.

Although the exact cause of preeclampsia remains unclear, previous research suggests that it is multifactorial, including obstetric, clinical, and sociodemographic factors [8,9,10]. Recent studies show that environmental factors, including air pollution, may play a more important role in the occurrence of this hypertensive disorder of pregnancy than previously thought. Air pollution is mainly caused by urbanization, vehicle emissions, and industrial activities, including the release of NO_2_, SO_2_, and PM [11,12]. Heavy metals are typically bound to particulate matter from these sources [13]. Air pollution may contribute to hypertensive disorders and placental hypoxia through vasoconstriction, inflammation, and oxidative stress [14,15]. Abnormal placentation is considered an important factor in the development of hypertensive disorders [16]. The pathophysiology of preeclampsia is most commonly explained using a two-stage model. The first stage involves decreased placental blood supply due to inadequate trophoblastic invasion of the maternal spiral arteries. The second stage is characterized by the release of biological factors from the ischemic placenta, causing endothelial injury and leading to acute maternal syndrome and systemic multiple organ failure [17,18].

The association between air pollution exposure and hypertensive disorders during pregnancy has been investigated by many studies, but the findings are not consistent [19,20,21].

This study aims to clarify the effect of air pollutants on hypertensive disorders of pregnancy by analyzing retrospective data from the National Health Insurance (NHI) Service in Korea and comparing the incidence of preeclampsia and eclampsia according to the air pollutant exposure.

## 2. Materials and Methods

*Background and Objectives*: Air pollutants have been shown to affect hypertensive disorders and placental hypoxia due to vasoconstriction, inflammation, and oxidative stress. The objective of this study was to evaluate whether high levels of maternal exposure to PM2.5 and heavy metals during the second trimester of pregnancy are associated with an increased risk of preeclampsia and eclampsia, using national health insurance claims data from South Korea. *Methods:* Data on mothers and their newborns from 2016 to 2020, provided by the National Health Insurance Service, were used (*n* = 1,274,671). Exposure data for ambient air pollutants (PM2.5, CO, SO_2_, NO_2_, and O_3_) and heavy metals (Pb, Cd, Cr, Cu, Mn, Fe, Ni, and As) during the second trimester of pregnancy were retrieved from the Korea Environment Corporation. The Korea Environment Corporation is a government agency of Korea and launched a website (https://www.airkorea.or.kr, last accessed on 18 April 2022) in 2005 to provide nationwide data on outdoor air quality. This agency operates over 500 measuring stations across Korea. Measurements of air pollutants are reported as a monthly average.

This study used air pollution monitoring data provided by the Korea Environment Corporation from 16 regions in South Korea on the monthly average concentrations of air pollutants from 2016 to 2020. The atmospheric condition data during the mothers’ respective pregnancy periods were matched based on their registration area and pregnancy periods, obtained from the National Health Insurance Service.

In another study that analyzed the same area, outdoor sources were the predominant contributors to particle exposure compared to indoor sources, because the daily integrated particle exposures were three times higher from outdoor than from indoor sources [22]. Moreover, pregnant women engaged in the highest levels of various types of physical activity during second trimester [23]. Therefore, we used atmospheric condition data without adjusting for indoor exposure.

A logistic regression model was adjusted for maternal age, infant sex, season of conception, and household income. *Results:* In total, 16,920 cases of preeclampsia and 592 cases of eclampsia were identified. In the multivariate model, copper exposure remained significantly associated with an increased risk of preeclampsia (odds ratio: 1.011; 95% confidence interval: 1.001–1.023), and higher ozone exposure during pregnancy was associated with an elevated risk of eclampsia. *Conclusions:* Increased copper exposure during the second trimester of pregnancy was associated with a high incidence of preeclampsia, whereas increased ozone exposure during the second trimester of pregnancy was associated with a high incidence of eclampsia. Measured data on ambient air pollutants (PM10, PM2.5, SO_2_, NO_2_, O_3_, and CO) and heavy metals (Pb, Cd, Cr, Cu, Mn, Fe, Ni, and As) in South Korea from January 2016 to December 2020 were extracted from the Korea Environment Corporation (https://www.airkorea.or.kr/eng, last accessed on 18 April 2022). Atmospheric conditions were matched to mothers and their newborns based on the mother’s NHI registration area. Air pollutant data measured during pregnancy were matched to the mother’s health insurance claim registration. The gestational period was divided into three stages: the first 1–3 months were defined as stage 1, 4–7 months as stage 2, and 8–10 months as stage 3. Household incomes were categorized, with the lowest 40% being defined as low-income and the highest 5% as high-income.

### Statistical Analysis

Data are presented as the mean ± standard deviation and the number (%) of patients. Confounding variables and key characteristics of the groups with and without preeclampsia and eclampsia were analyzed using an independent *t*-test for continuous variables and Fisher’s exact test or the chi-square test for categorical variables.

Logistic regression analysis was conducted to assess the association between exposure to air pollutants and heavy metals during the second trimester of pregnancy and the risks of preeclampsia and eclampsia. A single-pollutant model was applied to each pollutant, and univariate analyses were conducted to identify the associations.

Variables included in the multivariate model were selected based on their clinical relevance to preeclampsia and eclampsia, as well as their statistical significance in the univariate analysis. The model was adjusted for maternal age, infant sex, gestational age, and household income. Adjusted odds ratios (ORs) and 95% confidence intervals (CIs) for preeclampsia and eclampsia were calculated to evaluate the effects of second-trimester exposure to selected air pollutants and heavy metals.

All statistical analyses were performed using SAS^®^ version 9.4 (SAS Institute Inc., Cary, NC, USA), with statistical significance set at a *p*-value of less than 0.05.

## 3. Results

**Figure 1:**
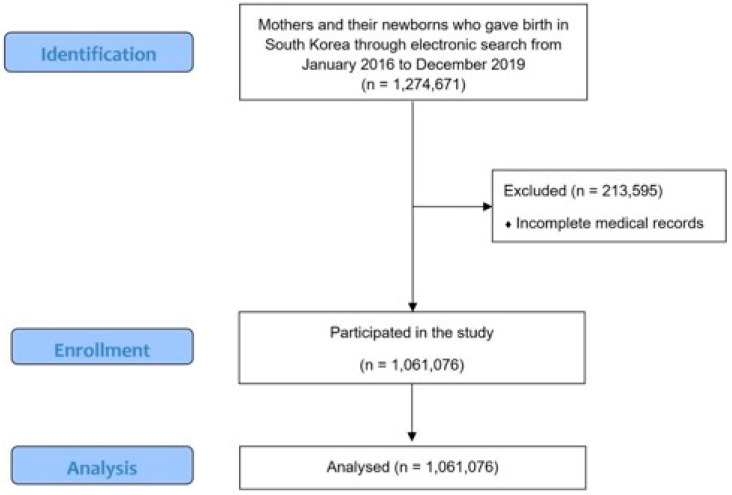
Consort flow diagram of this study.

From January 2016 to December 2020, data on 1,274,671 mothers and their newborns in South Korea were retrieved through an electronic search of NHI claim data. After excluding 213,595 cases due to incomplete medical records, a total of 1,061,076 mother–newborn pairs were included in the final analysis (Figure 1). The study population was analyzed to evaluate the effects of fine dust and air pollutant exposure during pregnancy on the incidence of preeclampsia and eclampsia.

Table 1 presents the demographic and clinical characteristics of the study participants, comparing individuals with preeclampsia and eclampsia to those without these conditions. Statistically significant differences (*p* < 0.05) were observed across several categories, including maternal age, household income, infant sex, and pre-existing health conditions. The rates of pre-existing hypertension, gestational hypertension, and gestational diabetes were significantly higher among participants with preeclampsia or eclampsia compared to those in the general study population.

**Figure 2:**
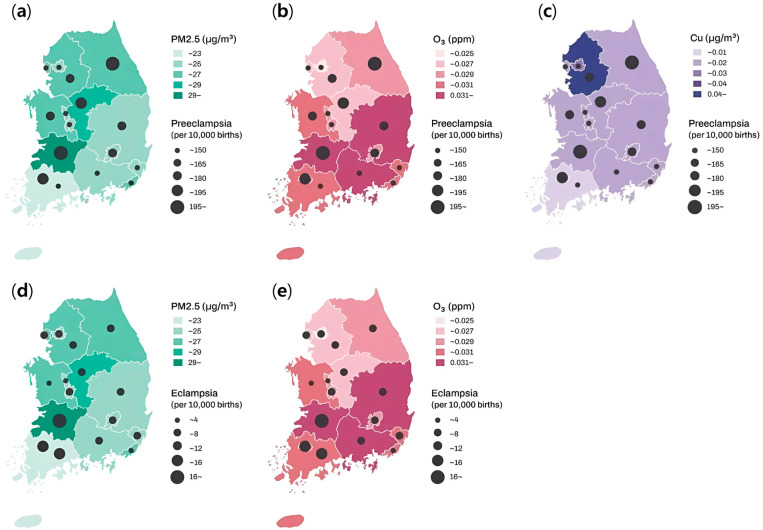
Spatial distribution of the mean PM2.5, O_3_, and Cu concentrations and the number of preeclampsia and eclampsia cases in South Korea. (**a**) PM2.5 and preeclampsia, (**b**) O_3_ and preeclampsia, (**c**) Cu and preeclampsia, (**d**) PM2.5 and eclampsia, (**e**) O_3_ and eclampsia. Data are presented as the mean concentration of each pollutant and the number of cases per 10,000 births.

Table 2 summarizes the statistics on air pollutants and heavy metals for each case study. Figure 2 presents the spatial distribution of the mean PM2.5, O_3_, and Cu concentrations, as well as the number of preeclampsia and eclampsia cases across South Korea. Higher mean concentrations of PM2.5 and O_3_ were associated with an increased incidence of both preeclampsia and eclampsia, whereas elevated Cu concentrations were specifically linked to an increased incidence of preeclampsia.

Table 3 shows the association between exposure to specific air pollutants and heavy metals during the second trimester and the incidences of preeclampsia and eclampsia. Exposure to PM2.5, O_3_, and Cu was significantly associated with an increased risk of preeclampsia. O_3_ exposure was also significantly associated with an increased risk of eclampsia.

Table 4 presents the association between exposure to PM2.5, O_3_, and Cu and the risk of preeclampsia. In the univariate analysis, PM2.5 exposure was significantly associated with an increased risk of preeclampsia. In the multivariate model, Cu exposure remained significantly associated with an increased risk of preeclampsia (OR: 1.011, 95% CI: 1.001–1.023), suggesting that it may be an independent risk factor.

Table 5 illustrates the association between PM2.5 and O_3_ exposure and the risk of eclampsia. In the univariate analysis, O_3_ exposure was significantly associated with an increased risk of eclampsia, and this association remained significant in the multivariate model (OR: 1.113; 95% CI: 1.007–1.23), suggesting that O_3_ may be an independent risk factor for eclampsia. These findings suggest that elevated O_3_ exposure during pregnancy is associated with a higher risk of eclampsia.

## 4. Discussion

This study demonstrated that Cu exposure during the second trimester of pregnancy could be an independent risk factor for preeclampsia, while O_3_ exposure during the same period might independently contribute to the risk of eclampsia. Increased Cu exposure during the second trimester was associated with a higher incidence of preeclampsia, whereas increased O_3_ exposure during the same period was linked to a higher incidence of eclampsia.

The adverse effects of air pollution on human health have garnered growing attention worldwide. Recently, more research institutions have begun investigating the associations among preeclampsia, eclampsia, and air pollution. However, these studies have produced conflicting results and often oversimplify the analysis of air pollutants. Air pollutants consist of complex components, including nitrogen dioxide, sulfur dioxide, particulate matter, indirectly formed ozone, and heavy metals. Heavy metals often amalgamate with particulate matter and primarily originate from diesel and gasoline exhaust emissions, particularly in areas with high traffic and industrial activity. South Korea is an appropriate country to investigate the effects of heavy metals in air pollution on pregnant women, because its geographical characteristics and rapid urbanization and industrialization have resulted in higher concentrations of heavy metals in air pollution than the global average [24,25,26].

We analyzed data from 16,920 women who were diagnosed with preeclampsia out of 1,061,076 deliveries in Korea between January 2016 and December 2020. The incidence rate of preeclampsia was 1.59%, which is lower than that reported in other studies [27]. This discrepancy may be associated with the exclusion of patients with incomplete medical records or the fact that the study population was primarily Asian [28].

We examined the association between exposure to air pollution during pregnancy and the risk of preeclampsia, focusing on exposure during the second trimester. Although the etiology of preeclampsia has not been fully elucidated, one of the most commonly accepted theories is the two-stage model. In stage I, inadequate trophoblast invasion leads to shallow placentation and poor uteroplacental perfusion. In stage II, this results in widespread endothelial dysfunction and systemic clinical manifestations [29]. It is believed that stage I occurs before gestational age of 12 to 20 weeks, corresponding to the late first trimester and early second trimester, and stage II occurs thereafter [30]. Also, preeclampsia would require delivery or the termination of pregnancy under certain circumstances. Especially when the patient has not reached term pregnancy, there is an increased risk of preterm birth or pregnancy termination. As a result, third trimester data may be subject to selection survival bias. One study reported that the late first and second trimesters are likely the most influential periods for air pollution exposure and the development of preeclampsia, with the highest risk being associated with PM2.5 exposure identified in the fourth month of pregnancy [31]. Another study found that exposure to air pollutants in the second trimester was associated with an increased risk of gestational hypertension [32]. Additionally, Jia et al. showed that in the second trimester, multiple air pollutants were identified as risk factors for preeclampsia, with new pollutants that were not risk factors in the first trimester emerging as additional risks, indicating that the second trimester is more vulnerable to preeclampsia than the first [33]. Therefore, these authors concluded that exposure to airborne pollution particles has the greatest effect during the second trimester of pregnancy.

We confirmed that the risk factors for preeclampsia include adolescent pregnancy, working outside the home, lower economic status, infant sex, cold season, low birth weight, low gestational age, nulliparity, and comorbidities. This study also showed a significant association between exposure to PM2.5 and copper in air pollution during pregnancy and the risk of preeclampsia. Additionally, there was a significant association between exposure to O_3_ in air pollution during pregnancy and the risk of eclampsia.

This study demonstrated a significant association between exposure to PM2.5 during pregnancy and preeclampsia. Although the extent of placental exposure to PM2.5 is unknown, a recent study provided evidence that inhaled PM is translocated from the lungs to the placenta [34]. Generally, exposure to ambient PM is related to the inhibition of phagocytosis, enhancement of inflammatory responses, and elevated oxidative stress [35]. Additionally, an in vitro study found that exposure to PM2.5 increases the production of interleukin 6, a proinflammatory cytokine, and decreases the production of hCG in human first-trimester trophoblast cells [36]. It is speculated that PM2.5 may contribute to pathological changes in the placenta associated with preeclampsia. Similarly to our study, several meta-analyses have reported that exposure to PM2.5 during pregnancy is correlated with an increased risk of preeclampsia [37,38]. However, these meta-analyses have generally found that exposure to PM2.5 during pregnancy is positively associated with the risk of preeclampsia, whereas our study did not find a statistically significant association. This discrepancy may be attributed to the fact that our study was conducted in Korea, where PM2.5 concentrations are relatively uniform across regions because of the country’s small geographical area, unlike meta-analyses encompassing diverse countries and regions. Another meta-analysis reported that the risk of preeclampsia from an increase in PM2.5 during pregnancy was not statistically significant [39]. However, a subsequent meta-analysis that included more eligible studies reported a statistically significant association between these two factors [38].

Regarding exposure to O_3_ during pregnancy, this study found a modest association with the risk of preeclampsia. Evidence suggests that exposure to O_3_ during pregnancy may increase the risk of preeclampsia. Exposure to O_3_ during pregnancy was associated with an increased sFlt-1/PIGF ratio, a characteristic feature of preeclampsia [40,41]. In addition, a study using murine models found that maternal O_3_ exposure leads to alterations in the placental extracellular matrix, which is crucial for angiogenesis and vascular development [42]. Jia et al. reported a significant association between exposure to O_3_ during pregnancy and the risk of preeclampsia [33]. However, similarly to our results, Cheng et al. found no association between exposure to O_3_ during pregnancy and the risk of preeclampsia [43]. These discrepant results may be explained by the different levels of O_3_ pollution in various countries, regions, and populations. Jin et al. reported relatively higher levels of O_3_ pollution compared to those in our study and that of Cheng et al. Furthermore, Cheng et al. reported a significant association between O_3_ exposure and an increased risk of preeclampsia in older mothers, in contrast to the results observed in mothers of all ages.

Our results showed that the adjusted odds ratio and 95% CI for the association between preeclampsia and exposure to NO_2_ or SO_2_ during pregnancy were 0.826 (0.72–0.947) and 0.337 (0.158–0.718), respectively. We do not believe that the odds ratio indicates negative associations between NO_2_, SO_2_, and preeclampsia. Several studies have shown conflicting data regarding the association between NO_2_, SO_2_, and preeclampsia. Jia et al. reported that only the highest exposure to SO_2_ was a risk factor for preeclampsia in the second trimester [33]. Yan et al. found that ambient SO_2_ was not significantly associated with preeclampsia risk [44]. Other studies have reported that the risk of preeclampsia increases with an increase in NO_2_ concentration [33,45]. These results could indicate that the relationships between NO_2_, SO_2_, and preeclampsia are not typical linear relationships. Jiang et al. reported that NO_2_ and SO_2_ show a non-linear relationship with preeclampsia in a concentration-dependent manner [46]. This suggests the need for more precise exposure modeling in future studies.

In areas with air pollution, due to the ubiquity of Cu in the environment and its presence in airborne particulates, exposure to Cu through inhalation is commonplace, particularly in environments with elevated Cu concentrations [47,48]. The mechanism through which copper, a component of air pollutants, contributes to preeclampsia development remains unclear. However, due to their extremely small size, copper nanoparticles may cross biological barriers, enter the bloodstream, and eventually accumulate in other organs [49]. In vitro studies using human cell lines have shown that copper nanoparticles exhibit DNA-damaging effects, potentially mediated by reactive oxygen species and oxidative stress [50,51]. Nanoparticles can interact with organelles such as mitochondria and induce the production of reactive oxygen species, disrupting cellular functions and leading to cytotoxicity, genotoxicity, inflammation, or apoptosis [52]. In vivo study have demonstrated that Cu nanoparticles increased the production of reactive oxygen species associated with serious adverse effects such as genotoxicity, inflammation, and apoptosis [53]. Tadesse et al., using in vivo and in vitro models, demonstrated that oxidative stress-induced DNA damage is present in higher amounts in the placentas of women with preeclampsia and appears to be clustered in the genomic DNA of maternal decidual stromal cells [54]. It is reasonable to suggest that such DNA damage could affect the placentation, thereby contributing to the pathogenesis of preeclampsia. Despite these findings, the effect of Cu on preeclampsia remains epidemiologically inconclusive. Several studies have reported that Cu is associated with the development of preeclampsia [55,56], whereas other studies have found that Cu is not associated with the development of preeclampsia [57,58] or is associated with a low preeclampsia risk [59,60]. A recent meta-analysis reported that the discrepancies among these studies may be explained by a U-shaped effect, suggesting that abnormally low or high levels of serum Cu can lead to a high risk of preeclampsia [61]. In our study, we found a statistically significant association between air pollutant Cu exposure and preeclampsia, although the effect size was modest. This may be explained by another study conducted in the same region as ours, which showed that serum Cu levels in pregnancies with preeclampsia were neither abnormally high nor low [62].

This study shows a significant association between O_3_ exposure during pregnancy and the risk of eclampsia. However, as eclampsia represents a severe progression of preeclampsia, this result should be interpreted with caution. The low incidence and small number of patients with eclampsia may have contributed to this finding; therefore further research is warranted.

This study has several limitations. One is that we could not adjust for the potential impact of heavy metals originating from sources other than airborne exposure. Heavy metals are present not only in the air but also in soil and water and can enter the human body through the oral route. However, previous studies analyzing heavy metals in food and water in the same areas as in our study have reported that their levels were within acceptable ranges or at low concentrations [62,63,64]. Therefore, the influence of heavy metals in soil or water is likely negligible. Another is that our study did not adjust for the interaction between PM2.5 and heavy metals and did not include confounding factors such as pre-pregnancy BMI and occupational exposure. Although PM2.5 constitutes a small percentage of suspended particles, its large surface area and ability to adsorb various chemicals may result in varying magnitudes of effect depending on its composition. Jeon et al. showed that health risks may differ across regions due to variations in PM2.5 component concentrations [65]. According to Chen et al., different components of PM2.5 affect distinct metabolic pathways [66]. Due to these complexities, we did not conduct an interaction analysis. We included all high-risk factors for preeclampsia, as well as some moderate and other risk factors, based on the classifications provided by the National Institute for Health and Care Excellence guidelines. However, several moderate or other risk factors such as pre-pregnancy BMI and occupational exposure were not included due to the absence of corresponding data in the database. Finally, the identification of disease status based on ICD diagnostic codes may result in misclassification, potentially introducing bias into the findings.

## 5. Conclusions

This study demonstrated that exposure to PM2.5 and Cu during the second trimester of pregnancy is significantly associated with an increased risk of preeclampsia. These findings underscore the vulnerability of pregnant women to air pollutants, especially during the second trimester, a critical period for placental and vascular development. Despite several limitations, our findings highlight a direct link between heavy metals in air pollution and the risk of preeclampsia.

## Figures and Tables

**Table 1:** Demographic and clinical characteristics of study participants.

	Total	With Preeclampsia	With Eclampsia
Maternal age (years)			
Total	1,061,076	16,920	592
<20 *†	203 (0.0)	9 (0.1)	1 (0.2)
20–30	107,623 (10.1)	1578 (9.3)	75 (12.7)
30–40	783,542 (73.8)	11,780 (69.6)	401 (67.7)
40<	169,708 (16.0)	3553 (21.0)	115 (19.4)
Occupational status *†			
Worked outside home	429,667 (40.5)	7191 (42.5)	272 (45.9)
Household income			
Low (40%) *	388,197 (36.6)	6811 (40.3)	234 (39.5)
Middle (40–95%)	649,430 (61.2)	9783 (57.8)	351 (59.3)
High (95%<)	23,449 (2.2)	326 (1.9)	7 (1.2)
Infant sex			
Female *†	516,809 (48.7)	8486 (50.2)	316 (53.4)
Season			
Winter *†	267,164 (25.2)	4353 (25.7)	152 (25.7)
Spring	279,390 (26.3)	4273 (25.3)	148 (25.0)
Summer	261,856 (24.7)	4048 (23.9)	137 (23.1)
Fall	252,666 (23.8)	4246 (25.1)	155 (26.2)
Body weight			
<1 kg *†	2060 (0.2)	442 (2.6)	11 (1.9)
1–2.5 kg	35,697 (3.4)	4038 (23.9)	109 (18.4)
2.5 kg<	1,023,319 (96.4)	12,440 (73.5)	472 (79.7)
Premature			
<28 weeks *†	1661 (0.2)	192 (1.1)	5 (0.8)
28–36 weeks	41,341 (3.9)	4614 (27.3)	120 (20.3)
36 weeks<	1,018,074 (95.9)	12,114 (71.6)	467 (78.9)
Multiple birth			
Singleton pregnancy *†	1,039,880 (98.0)	15,801 (93.4)	557 (94.1)
Twin pregnancy	20,433 (1.9)	1072 (6.3)	32 (5.4)
Multiple pregnancy	763 (0.1)	47 (0.3)	3 (0.5)
Pre-existing hypertension *†	37,236 (3.5)	5759 (34.0)	229 (38.7)
Gestational hypertension *†	5508 (0.5)	1003 (5.9)	56 (9.5)
Superimposed preeclampsia *†	1485 (0.1)	600 (3.5)	18 (3.0)
Pre-existing diabetes *†	2877 (0.3)	242 (1.4)	10 (1.7)
Gestational diabetes *†	229,918 (21.7)	5026 (29.7)	157 (26.5)
Kidney disease *†	6438 (0.6)	373 (2.2)	15 (2.5)
Autoimmune disease *	594 (0.1)	16 (0.1)	0 (0.0)
Thyroid disease *†	374,703 (35.3)	7683 (45.4)	267 (45.1)
Dyslipidemia *†	221,737 (20.9)	6226 (36.8)	234 (39.5)
Infertility *†	157,150 (14.8)	4210 (24.9)	134 (22.6)

Data are presented as the number (%) of patients. * indicates *p* < 0.05 for comparison between groups with and without preeclampsia. † indicates *p* < 0.05 for comparison between groups with and without eclampsia.

**Table 2:** Summary statistics of air pollutants and heavy metals by case.

Air Pollutants and Heavy Metals	Mean	SD
PM10 (μg/m^3^)	46.293	11.113
PM2.5 (μg/m^3^)	25.587	5.564
SO_2_ (ppm)	0.005	0.001
NO_2_ (ppm)	0.023	0.007
O_3_ (ppm)	0.027	0.008
CO (ppm)	0.503	0.110
Pb (μg/m^3^)	0.026	0.013
Cd (μg/m^3^)	0.001	0.001
Cr (μg/m^3^)	0.004	0.003
Cu (μg/m^3^)	0.025	0.014
Mn (μg/m^3^)	0.032	0.015
Fe (μg/m^3^)	0.664	0.267
Ni (μg/m^3^)	0.005	0.002
As (μg/m^3^)	0.004	0.002

**Table 3:** Adjusted odds ratios and 95% confidence intervals (CIs) for the association between preeclampsia or eclampsia and exposure to air pollutants and heavy metals during the second trimester of pregnancy, using a single-pollutant model.

	With Preeclampsia	With Eclampsia
	OR (95% CI)	OR (95% CI)
PM10	0.995 (0.993–0.997)	1.003 (0.993–1.013)
PM2.5	1.004 (1–1.008)	1.017 (0.997–1.037)
SO_2_	0.232 (0.201–0.268)	0.337 (0.158–0.718)
NO_2_	0.916 (0.893–0.94)	0.826 (0.72–0.947)
O_3_	1.05 (1.021–1.081)	1.312 (1.132–1.521)
CO	0.995 (0.993–0.997)	0.998 (0.988–1.008)
Pb	0.949 (0.936–0.962)	0.894 (0.831–0.962)
Cd	0.845 (0.693–1.031)	0.17 (0.048–0.607)
Cr	1.036 (0.98–1.095)	0.541 (0.387–0.755)
Cu	1.013 (1.002–1.024)	0.968 (0.913–1.027)
Mn	0.979 (0.968–0.989)	0.891 (0.837–0.948)
Fe	1 (0.999–1)	0.996 (0.992–0.999)
Ni	0.977 (0.917–1.042)	0.49 (0.341–0.705)
As	0.958 (0.897–1.024)	0.693 (0.476–1.009)

The logistic regression model is adjusted for maternal age, infant sex, and season of conception.

**Table 4:** Logistic regression analysis of risk factors for preeclampsia.

	Univariate	Multivariate
	OR (95% CI)	OR (95% CI)
Pre-existing hypertension	15.187 (14.663–15.731)	15.158 (14.643–15.692)
Twin and multiple pregnancy	2.541 (2.372–2.723)	2.537 (2.368–2.718)
Pre-existing diabetes and gestational diabetes	1.305 (1.26–1.351)	1.312 (1.267–1.358)
Age	1.001 (0.997–1.004)	
Kidney disease	1.482 (1.32–1.665)	1.483 (1.321–1.664)
Autoimmune disease	1.221 (0.727–2.051)	
Infertility	1.499 (1.442–1.558)	1.506 (1.45–1.564)
PM2.5	1.003 (1.001–1.006)	
O_3_	1.005 (0.985–1.025)	1.003 (0.984–1.023)
Cu	1.006 (0.995–1.018)	1.011 (1.001–1.023)

The logistic regression model is adjusted for maternal age, infant sex, and season of conception.

**Table 5:** Logistic regression analysis of risk factors for eclampsia.

	Univariate	Multivariate
	OR (95% CI)	OR (95% CI)
Pre-existing hypertension	16.549 (13.934–19.654)	16.538 (13.984–19.558)
Twin and multiple pregnancy	2.065 (1.447–2.947)	2.056 (1.441–2.933)
Pre-existing diabetes and gestational diabetes	1.102 (0.918–1.324)	
Age	0.984 (0.966–1.002)	
Kidney disease	1.433 (0.837–2.453)	
Infertility	1.318 (1.076–1.613)	1.303 (1.067–1.593)
PM2.5	1.014 (0.998–1.029)	1.013 (0.997–1.028)
O_3_	1.111 (1.005–1.228)	1.113 (1.007–1.23)

The logistic regression model is adjusted for maternal age, infant sex, and season of conception.

## Data Availability

Since the database is managed by the government, analysis is restricted to designated premises, and data extraction is not permitted.

## Abbreviations

The following abbreviations are used in this manuscript:

PM2.5	Particulate matter
NHI	National Health Insurance
ORs	Odds ratios
CIs	Confidence intervals
